# CAMKV Is a Candidate Immunotherapeutic Target in *MYCN* Amplified Neuroblastoma

**DOI:** 10.3389/fonc.2020.00302

**Published:** 2020-03-06

**Authors:** Robyn T. Sussman, Jo Lynne Rokita, Kevin Huang, Pichai Raman, Komal S. Rathi, Daniel Martinez, Kristopher R. Bosse, Maria Lane, Lori S. Hart, Tricia Bhatti, Bruce Pawel, John M. Maris

**Affiliations:** ^1^Division of Oncology and Center for Childhood Cancer Research, Children's Hospital of Philadelphia, Philadelphia, PA, United States; ^2^Department of Biomedical and Health Informatics, Children's Hospital of Philadelphia, Philadelphia, PA, United States; ^3^Department of Pathology, Children's Hospital of Philadelphia, Philadelphia, PA, United States; ^4^Department of Pathology, Children's Hospital of Los Angeles, Los Angeles, CA, United States

**Keywords:** CAMKV, *MYCN*, immunotherapy, neuroblastoma, ChIP-Seq

## Abstract

We developed a computational pipeline designed to use RNA sequencing (*n* = 136) and gene expression profiling (*n* = 250) data from neuroblastoma tumors to identify cell surface proteins predicted to be highly expressed in *MYCN* amplified neuroblastomas and with little or no expression in normal human tissues. We then performed ChIP-seq in the *MYCN* amplified cell lines KELLY, NB-1643, and NGP to identify gene promoters that are occupied by MYCN protein to define the intersection with the differentially-expressed gene list. We initially identified 116 putative immunotherapy targets with predicted transmembrane domains, with the most significant differentially-expressed of these being the calmodulin kinase-like vesicle-associated gene (CAMKV, *p* = 2 × 10^−6^). CAMKV encodes a protein that binds calmodulin in the presence of calcium, but lacks the kinase activity of other calmodulin kinase family members. We confirmed that CAMKV is selectively expressed in 7/7 *MYCN* amplified neuroblastoma cell lines and showed that the transcription of *CAMKV* is directly controlled by MYCN. From membrane fractionation and immunohistochemistry, we verified that CAMKV is membranous in *MYCN* amplified neuroblastoma cell lines and patient-derived xenografts. Finally, immunohistochemistry showed that CAMKV is not expressed on normal tissues outside of the central nervous system. Together, these data demonstrate that CAMKV is a differentially-expressed cell surface protein that is transcriptionally regulated by MYCN, making it a candidate for targeting with antibodies or antibody-drug conjugates that do not cross the blood brain barrier.

## Introduction

Neuroblastoma is an embryonal tumor that accounts for 12% of childhood cancer-related mortality (1, 2). Prognosis for low- and intermediate-risk neuroblastoma is outstanding with the majority of patients cured, but there has been minimal progress for patients with high-risk disease. About half of all high-risk neuroblastoma patients die despite intensive multimodal chemoradiotherapy (2). Recently, clinical trials of GD2-targeting monoclonal antibody therapy have demonstrated significantly prolonged relapse free survival when used after achieving a first response to standard therapy, leading to regulatory approval in the United States and Europe (3). However, patients suffer significant pain during dinutuximab infusions due to GD2 expression on pain fibers, limiting dosing and in part explaining minimal improvements in overall survival with this immunotherapy. Our understanding of how to develop successful immunotherapies has advanced significantly in the last few years, but the limiting factor remains identification of cell surface molecules uniquely and uniformly expressed on tumor cells.

Focal amplification of *MYCN* occurs in roughly 40–50% of high-risk neuroblastoma cases (4–6) and is associated with an aggressive phenotype and poor prognosis (2, 7). *MYCN* encodes a basic helix-loop-helix transcription factor that functions in transcription activation when heterodimerized with MAX, or transcriptional repression when heterodimerized with MNT, MXI, MAD, or other negative co-factors by binding to E-boxes within gene promoters (8, 9). Gene-expression profiling has revealed a large cohort of genes involved in cell cycle, proliferation, signaling, adhesion, differentiation, and migration to be regulated by MYCN (10–12). However, while *MYC* family genes are known to transcriptionally regulate a very large number of genes via enhancer invasion (13), surprisingly little is known about direct MYCN target genes.

While *MYCN* amplification is prevalent in high-risk neuroblastoma and some other pediatric cancers, and is an important biomarker for patient outcomes, it remains an elusive drug target. While direct targeting of the MYCN transcription factor is not yet possible, several indirect methods have been proposed such as depleting MYCN protein levels with BET or AURKA inhibitors (14–17), but these appear to be with limited anti-tumor efficacy. Here, we pursue another indirect strategy, identification of direct MYCN transcriptional targets that are located in the plasma membrane and thus amendable to new immunotherapeutic strategies.

## Methods

### Cell Lines and Chemicals

Cell lines were grown and STR validated as described (18–20). Cell lines were tested for mycoplasma when thawed and only grown for 20 passages following thaw. SHEP-2 MYCN-ER, and SK-N-AS MYCN-ER cells were obtained from the laboratory of Dr. Michael Hogarty at the Children's Hospital of Philadelphia. Cells were treated with 1 uM tamoxifen (Sigma h7904) to induce MYCN-ER nuclear translocation.

### Lentiviral Preparation and Transduction

Lentiviral preparation was carried out as described (21). Briefly, using the clone TRCN0000020695 to deplete MYCN (Sigma), plasmids encoding shRNA along with the envelope encoding plasmid pMD2.G and packaging plasmid psPAX2 were transfected into 293T cells with Fugene 6 (Roche). Supernatant was collected 48 and 72 h later, filtered and added to IMR-05 cells in the presence of 8 ug/ml polybrene (Sigma). Puromycin (Sigma) was used to select for infected cells.

### qRT-PCR

Total RNA was isolated from neuroblastoma cells utilizing RNeasy mini spin kits (Qiagen) and mRNAs were converted to cDNA using SuperScript II First Strand Synthesis kits (Life Technologies). *CAMKV* expression was detected using a Taqman probe (Hs01062060_g1, ThermoFisher) and *MYCN* was detected using (Hs00232074_m1, ThermoFisher), according to the methods previously described (19, 21).

### ChIP-qPCR

Chromatin immunoprecipitation was performed as previously described (22) using anti-MYCN (Santa Cruz Biotechnology, Inc., clone B8.4B, sc-53993), anti-MAX (Santa Cruz Biotechnology, Inc., clone H-2, sc-8011) and anti-mouse IgG (Santa Cruz Biotechnology, Inc., sc-2025). Primer sequences are as follows: CAMKV TSS Forward: 5′-GGGCAGAATCCGCTCCGA-3′;

CAMKV TSS Reverse: 5′-GCGATGCTGGAGGTTCGCTA-3′;

CAMKV 5′ Forward: 5′-CAAAGTCTCCTATCCCACCCC-3′;

CAMKV 5′ Reverse: 5′-TTTGGGAAAGACTCTGGGCTT-3′.

### ChIP-Seq

#### Chromatin Immunoprecipitation-Discovery Cohort

Chromatin immunoprecipitation was performed on the neuroblastoma cell lines Kelly, NB-1643 and NGPs as described (19, 23, 24). Briefly, using anti-MYCN (Santa Cruz B8.4B, sc-53993). Cells were grown to 80% confluence on 150 mm tissue culture plates in 20 mL of medium. Four hundred and fifteen microliters of 37% formaldehyde (final concentration 0.75% w/v) were added to the medium and the plate was rocked for 10 min to fix cells. 1.5 mL of 2.5 M glycine (final concentration 0.18 M) was added to quench the formaldehyde and the plate was rocked for an additional 5 min. Cells were lysed in 5 pellet volumes of FA lysis buffer (50 mM HEPES pH 7.5, 140 mM NaCl, 1 mM EDTA pH 8.0, 1.0% v/v Triton-X-100, 0.1% w/v SDS, 0.1% w/v sodium deoxycholate), supplemented with fresh protease inhibitors (Thermo Scientific, 88666) and 1 mM DTT. Beads were washed 3X in ChIP Wash Buffer (0.1% w/vSDS, 1.0% v/v Triton-X-100, 2 mM EDTA pH 8.0, 150 mM NaCl, 20 mM Tris-HCL pH 8.0) and once with Final Wash Buffer (0.1% w/v SDS, 1.0% v/v Triton-X-100, 2 mM EDTA pH 8.0, 500 mM NaCl, 20 mM Tris-HCL pH 8.0). Libraries were constructed using NEB ultra kit according to the manufacturer's instructions and sequenced on a MiSeq to a depth of ~50 M reads.

#### Chromatin Immunoprecipitation-Validation Cohort

The neuroblastoma cell lines COG-N-415, LA-N-5, NB-1643, NB-69 and SK-N-SH were grown to 60–80% confluence in a 150 mm dish, fixed with 11% w/v formaldehyde for 15 min, and quenched with 2.5 M glycine for 5 min at room temperature. Cells were scraped, washed with 10 mL of chilled PBS with 0.5% v/v Igepal (Sigma #1-8896), and pelleted. Supernatant was removed and cells were washed with 10 mL of chilled PBS, 0.5% Igepal, and 100 uL 100 mM PMSF. Cells were pelleted, supernatant removed, and sent to Active Motif on dry ice for chromatin immunoprecipitation. N-Myc and c-Myc ChIP reactions were performed using 30 μg of cell line chromatin and 6 μg of N-Myc antibody (Active Motif, cat # 61185) or 4 μg of c-Myc antibody (Santa Cruz N262, cat # sc-764). Libraries were prepared according to www.activemotif.com/services and sequenced on a NextSeq 500 to a depth of ~50 M reads.

#### Chromatin Immunoprecipitation-Analysis

Phred sequencing scores (25) were calculated for each sample using a perl script (https://raw.githubusercontent.com/douglasgscofield/bioinfo/master/scripts/phredDetector.pl) for automated input into the alignment algorithm. Quality was assessed using FastQC (http://www.bioinformatics.babraham.ac.uk/projects/fastqc/) and sequences were adapter- and quality-trimmed using Trim Galore (26, 27). Following QC, bwa (28) was used to align the reads to hg 19 reference genome and Picard tools (29) was used to remove duplicates. Fragment sizes were estimated using MaSC (30) and these were used as input into MACS2 (31) for peak calling. Peaks were called significant using a *q*-value (minimum False Discovery Rate) cut off of 0.05. Results were returned in units of signal per million reads to get normalized peak values.

Finally, repetitive centromeric, telomeric, and satellite regions known to have low sequencing confidence were removed using the blacklisted regions defined by UCSC.

#### Chromatin Immunoprecipitation-Data Availability

Kelly and NGP MYCN ChIP-Seq have been published under GEO accession number GSE94782 and the remaining MYCN and MYC ChIP-Seq data are available under GEO accession number GSE138295.

### Immunofluorescence

Cells were washed with cold PBS and fixed with a 50% Acetone/ 50% Methanol mixture for 5 min at −20°C. Cells were blocked with 0.3% v/v Triton X-100, 2% v/v goat serum in PBS for 5 min and then incubated with primary antibody in TBS-BGT (25 mM Tris, pH 8.0, 137 mM NaCl, 3 mM KCL, 1.5 mM MgCl_2_, 5 mg/ml BSA, 1 mg/ml Glycine, 0.05% v/v Tween 20, 0.02% v/v sodium azide) for 90 min at room temperature. Primary antibodies included m906IgG (32) and anti-CAMKV (Santa Cruz Biotechnology, Inc., clone S-17, sc-102408). Cells were then washed with TBS-BGT and incubated with secondary antibody in TBS-BGT. Cells were washed again in TBS-BGT and mounted to slides with prolong gold with DAPI (ThermoFisher Scientific, P36931).

### Cell Plugs

10–20 × 10^6^ live neuroblastoma cells were suspended in 50 uL of medium. A 1% w/v agarose in PBS solution was boiled and cooled to 50°C in a water bath. The cell suspension was mixed with 0.5 mL of the agarose and transferred into an inverted capped Eppendorf tube with the conical bottom cut off, on ice. Once solidified, the plug was removed, cut in half, and fixed in 10% v/v buffered formalin (ThermoFisher Scientific).

### Immunocytochemistry

CAMKV staining was performed using the CAMKV S-17 antibody (Santa Cruz Biotechnology, Inc.). Each sample was scored by the same pathologist and was designated as “1” when <10% of cells stained positively, “2” when 10–90% of tumors stained positively and “3” when >90% of cells stained positively. Only cells of targeted tissue type were evaluated for NCAM staining.

### Western Blotting

Cells were lysed and western blotting was performed as previously described (18) using the following antibodies: anti-CAMKV (Santa Cruz Biotechnology, Inc., sc-102408) was used at a 1:250 dilution, anti-ACTIN (Cell Signaling, 4967s) was used at a 1:5,000 dilution, anti-ADAM12 (abcam, ab28225) was used at 1:1,000 and anti-BCL-2 (Cell Signaling, 2870S) was used at a 1:2,000 dilution.

### Membrane Isolation

Membrane fractionation was performed using the Proteoextract Native Membrane Protein Extraction Kit (Millipore, 444810) according to the manufacturer's instructions.

### RNA-seq Expression Analysis

RNA sequencing data from 150 primary neuroblastoma tumors (136 high risk) was generated by the Therapeutically Applicable Research to Generate Effective Treatments project (TARGET data matrix, http://ocg.programs/target/data-matrix). RNA sequencing data were obtained from The Cancer Genome Atlas Program (TCGA), the Children's Brain Tumor Tissue Consortium (CBTTC), the Pacific Pediatric Neuro-Oncology Consortium (PNOC), the GEO database (GSE60052), and the Genotype-Tissue Expression (GTEx) project. Gene level data was generated using STAR alignment and RSEM normalization using hg 38 as a reference genome and GENCODE V23 gene annotation (33). The voom procedure was used to normalize the RSEM generated expected counts followed by differential expression testing using the R package limma to obtain adjusted *p*-values and Log-fold changes (LogFCs). Tumors were stratified by *MYCN* expression and sequencing data was queried for genes that had a LogFC >1 in the *MYCN* amplified tumors compared to non-amplified tumors with an adjusted *p*-value of 0.05 by the Benhanini-Hochberg procedure.

### Gene-Expression Array Analysis

Gene expression data generated using Human Exon arrays (Affymetrix) from 250 primary tumors were obtained from the TARGET data matrix. These data were processed with Robust Mutlichip Average (RMA) normalization analysis implemented in the Affymetrix Power Tools (Affymetrix, Inc.). Samples were stratified by *MYCN* expression and the top and bottom 15% from this stratification were then used to query for the most differentially overexpressed genes in the *MYCN*-high subset of tumors.

### Plasma Membrane Designation

The COMPARTMENTS database (http://compartments.jensenlab.org) was used to determine association with the plasma membrane (34). We designated only those genes to be potentially associated with the plasma membrane where the max confidence score across all GO categories was associated with Plasma Membrane or Cell Surface and was ≥3.

## Results

### Identification of Plasma Membrane Proteins Specifically Over-expressed in MYCN Amplified Neuroblastomas

**Figure 1:**
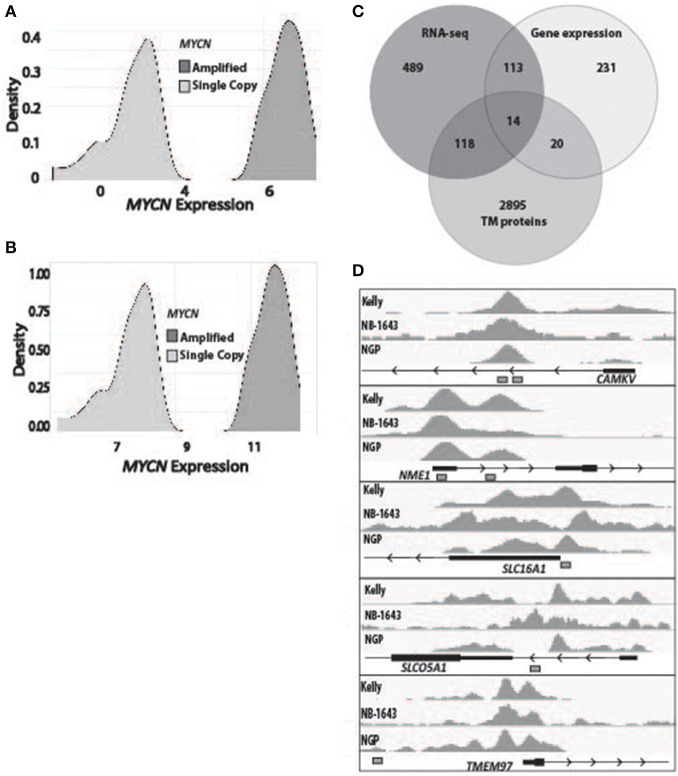
Identification of transmembrane-domain containing MYCN target genes in *MYCN* amplified neuroblastoma. **(A)** RNA sequencing data from 150 primary neuroblastoma tumors were stratified by *MYCN* expression and queried for the most differentially overexpressed genes (Log fold change >1, adjusted *p*-value of 0.05) in the *MYCN* high tumors. **(B)** Gene expression data from 250 primary neuroblastoma tumors were stratified by *MYCN* expression and queried for the most differentially overexpressed genes in the *MYCN* high tumors. **(C)** The most differentially overexpressed genes from the *MYCN* high tumors from RNA-sequencing data and gene expression data were intersected with a list of genes that produce transmembrane proteins (34). **(D)** Chromatin Immunoprecipitation sequencing tracks from five targets that were bound by MYCN protein at transcription start site-proximal E-boxes (depicted by white boxes) in the *MYCN* amplified Kelly, NB-1643 and NGP cell lines.

To determine the subset of genes which were differentially overexpressed in *MYCN* amplified neuroblastoma, we first used RNA-sequencing data from 150 primary tumors (136 of which were high-risk) and stratified them by *MYCN* expression (Figure 1A). We then filtered for genes that had a log fold change over 1 in the *MYCN* amplified tumors compared to non-amplified tumors with an adjusted *p*-value of 0.05. We similarly analyzed microarray gene expression data from 250 primary neuroblastomas stratified by *MYCN* expression (Figure 1B). The top and bottom 15% from this stratification were then used to query for the most differentially overexpressed genes in the *MYCN*-high subset. We intersected these data with a list of genes predicted to encode proteins located in the plasma membrane by the COMPARTMENTS database (34) and found 14 to be consistently differentially overexpressed in the *MYCN* amplified tumors (Figure 1C, Table 1, Table S1).

**Table 1:** Differentially overexpressed genes in MYCN high tumors with transmembrane domains.

**Gene**	**Microarray** **LogFC**	**Microarray adj.** **p value**	**RNAseq** **LogFC**	**RNAseq adj.** ***p*-value**	**TM list?**	**E-Box near** **TSS?**	**MYCN bound near TSS?**	**E-box occupied by MYCN?**	**Limited normal tissue expression?**	**Not secreted?**
*CAMKV*	1.47	1.04E-06	1.73	2.02E-06	X	X	X	X	X	X
*CPNE7*	1.38	1.32E-09	2.75	1.33E-07	X	X	X	X		
*GLRA2*	1.52	0.0013487	2.80	1.27E-05	X	X				
*HHIP*	1.38	1.52E-05	1.89	2.05E-06	X	X	X	X		
*KCNH5*	1.45	0.0001737	2.11	0.005154	X	X	X			
*LGR5*	1.26	0.000363	2.38	1.43E-06	X					
*TMEM131L*/ *KIAA0922*	1.06	3.31E-06	1.05	4.23E-11	X	X	X	X		
*LRRC7*	1.29	0.0017533	2.02	2.54E-05	X	X	X	X	X	
*NME1*		2.00E-08	1.00	1.17E-06	X	X	X	X	X	X
*SLC30A3*	1.10	6.26E-08	4.06	2.64E-10	X					
*SLC16A1*	1.37	1.06E-07	1.08	9.40E-10	X	X	X	X		
*SLCO5A1*	1.84	8.96E-13	2.08	3.93E-10	X	X	X	X		
*TMEM97*	1.89	3.49E-11	1.56	9.48E-14	X	X	X			
*SLC44A5*	1.42	1.01E-05	1.33	0.002541	X					

To prioritize these 14 candidate immunotherapeutic targets, we first surveyed for transcription start site (TSS)-proximal E-box motifs, and showed that these were present in 11 (Table 1). To determine which of these genes are transcriptionally dependent on MYCN being bound to their promoters, we performed ChIP-seq in the *MYCN* amplified cell lines NB-1643, NGP, and Kelly (discovery cohort). We later performed additional MYCN ChIP-seq in the *MYCN* amplified cell lines COG-N-415, LA-N-5, and repeated NB-1643, as well as the non-amplified cell line NB-69 (validation cohort). Ten of these 11 differentially overexpressed gene loci were occupied by MYCN, 8 at transcription start site-proximal E-boxes (Figure 1D, Figure S1, Table 1). We next looked at the normal expression of these genes and eliminated any with widespread normal tissue expression. For this analysis, we included genes that had normal tissue expression limited to the central nervous system, considering larger biologics such as antibodies would not cross the blood-brain barrier (35), reducing our gene list to 3 (Table 1). To ensure that the protein products of these genes were not only expressed at the membrane, but were membrane bound and able to be targeted with a therapeutic, we eliminated genes with secreted products. Of the remaining two genes, *NME1* has been described for its prognostic value in neuroblastoma and mutations in *NME1* correlate with an aggressive phenotype (36, 37). While *NME1* is somewhat differentially overexpressed in *MYCN* amplified neuroblastoma (logFC = 1, adjusted *p*-value = 1.17 × 10^−6^, Table S1) the protein product of *NME1*, nm23-H1, has diffuse sub-cellular localization that does not always include the plasma membrane (38) and mostly consists of the cytosol and endoplasmic reticulum (39), suggesting it would not be an ideal candidate for targeted immunotherapy. *Calmodulin kinase-like vesicle associated (CAMKV)* is more significantly differentially overexpressed in *MYCN* amplified tumors than *NME1* (logFC = 1.73, adjusted *p*-value = 2.02 × 10^−6^). CAMKV has limited normal tissue expression in the brain (Figure S2) and has a TSS-proximal E-box that is occupied by MYCN protein. While we had intended to nominate candidate immunotherapy targets for *MYCN* amplified neuroblastoma that lacked normal tissue expression, the analysis did not yield any targets. Thus, we expanded our analysis to include normal expression on CNS tissues with the goal of using an antibody-based therapeutic that wouldn't cross the blood-brain barrier. From this analysis, we nominated the unstudied protein CAMKV as a putative immunotherapeutic target for *MYCN* amplified neuroblastoma.

**Figure 2:**
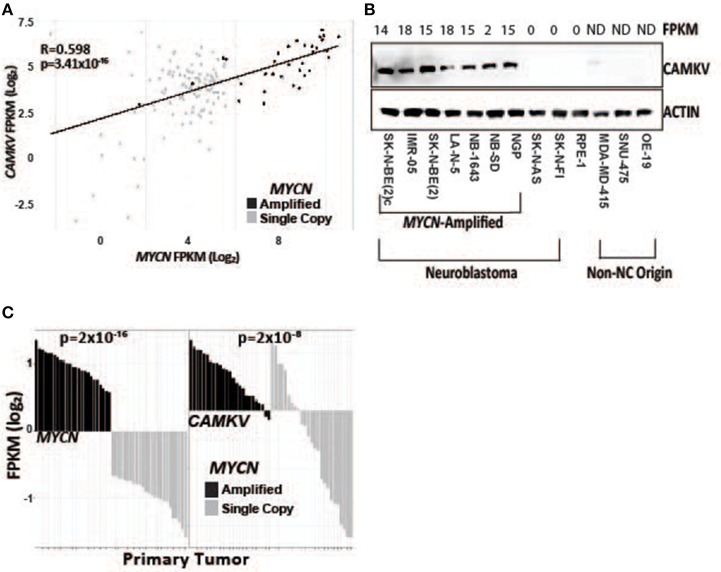
*CAMKV* expression correlates with *MYCN* expression. **(A)** *MYCN* and *CAMKV* FPKM correlate across 150 primary neuroblastoma tumors (R = 0.598, *p* = 3.41 × 10^−16^). **(B)** *CAMKV* is expressed in *MYCN* amplified neuroblastoma cell lines (lines 1–7) but not non-amplified lines (8, 9), retinal pigment epithelial cells (9) or non-neural-crest origin cells (11–13). FPKM for *CAMKV* in cell lines are shown at top, ND = not detected. **(C)** RNA sequencing data from 150 primary neuroblastoma tumors were stratified by *MYCN* expression and the FPKM for *MYCN* (left) and *CAMKV* (right) for each tumor are plotted. Tumors with amplified *MYCN* are colored in black and non-amplified tumors are gray. *MYCN* amplification correlates with *MYCN* FPKM (*p* = 2 × 10^−16^) and *CAMKV* FPKM (*p* = 2 × 10^−8^).

To further explore the association of *CAMKV* and *MYCN* co-expression, we stratified RNA-sequencing and gene expression data from primary tumors by *MYCN* amplification status. We performed correlation analysis in primary tumors between *MYCN* and *CAMKV* and found them to be significantly correlated (Figures 2A,C). Finally, we examined if *CAMKV* protein expression is selectively expressed in *MYCN* amplified neuroblastoma cell lines. Whole cell lysate from 7 *MYCN* amplified neuroblastoma cell lines was compared to two non-amplified neuroblastoma cell lines, immortalized retinal pigment epithelial cells, and three non neuroblastoma, non-neural crest origin cancer cell lines and found *CAMKV* protein to be exclusively expressed in the *MYCN*-amplified neuroblastoma cell lines (Figure 2B). Together, these data suggest that *MYCN* amplification is a predictive biomarker of high *CAMKV* mRNA and protein expression.

**Figure 3:**
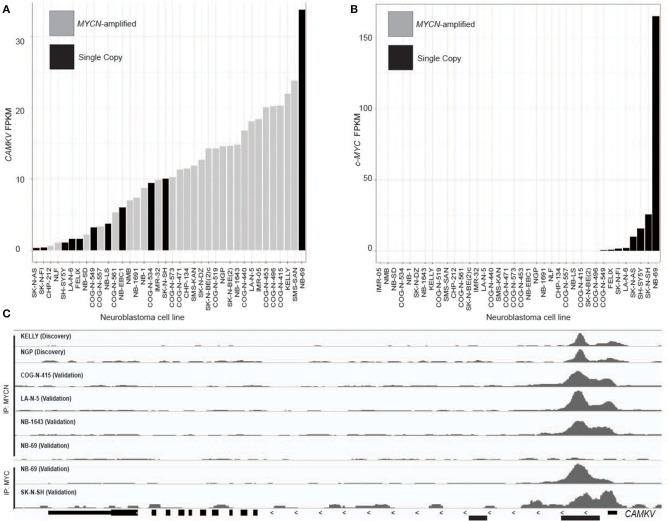
*CAMKV* is expressed in a limited number of non-amplified tumors with high MYC. **(A)** Neuroblastoma cell lines in rank order of *CAMKV* expression. *MYCN* amplified tumors are gray and single copy tumors are black. **(B)** Neuroblastoma cell lines in rank order of c-MYC expression. *MYCN* amplified tumors are gray and single copy tumors are black. **(C)** Chromatin immunoprecipitation sequencing tracks at the *CAMKV* locus from the *MYCN* amplified cell lines Kelly, NGP, COG-N-415, LA-N-5, NB-1643, and non-amplified lines NB-69 and SK-N-SH. Cells were immunoprecipitated using either an anti-MYCN antibody (top tracks) or an anti-MYC antibody (bottom tracks).

A limited number of non-amplified tumors appeared to have high *CAMKV* expression (Figures 2A,C). A recently defined subset of high-risk neuroblastoma tumors that lack *MYCN* amplification overexpress the MYC protein and have a poor prognosis comparable to those with amplified *MYCN* (40–42). While the majority of *CAMKV*-expressing cell lines are *MYCN* amplified (Figure 3A), the highest CAMKV-expressing cell line is NB-69 which does not harbor *MYCN* amplification, but has the highest *MYC* expression (Figure 3B). To further interrogate how MYCN and/or MYC protein(s) control *CAMKV* expression, we performed validation CHIP-sequencing for MYCN protein in the *MYCN* amplified neuroblastoma cell lines: COG-N-415, LA-N-5, NB-1643, and for MYC protein in the *MYCN* non-amplified lines: NB-69 and SK-N-SH (Figure 3C). We additionally performed MYCN ChIP-Seq on the non-amplified NB-69 cell line as a negative control. The *MYCN* amplified cell lines COG-N-415, LA-N-5, and NB-1643 all show MYCN occupancy at the *CAMKV* TSS-proximal E-boxes, similar to KELLY and NGP from the discovery cohort (top two tracks). The NB-69 cell line does not harbor *MYCN* amplification and has no MYCN occupancy at the TSS-proximal E-box; NB-69 is the highest *MYC-* and *CAMKV*-expressing cell line, and indeed shows MYC occupancy at the *CAMKV* TSS-proximal E-box (Figure 3C). The SK-N-SH cell line has the second highest *MYC* expression after NB-69, the second-highest *CAMKV* expression among non-amplified cell lines, and also has MYC occupancy at the CAMKV TSS-proximal E-box (Figure 3C). Together, these data suggest that in some high-risk neuroblastomas that lack *MYCN* amplification, MYC can drive *CAMKV* transcription.

### MYCN Protein Directly Controls the Transcription of CAMKV

**Figure 4:**
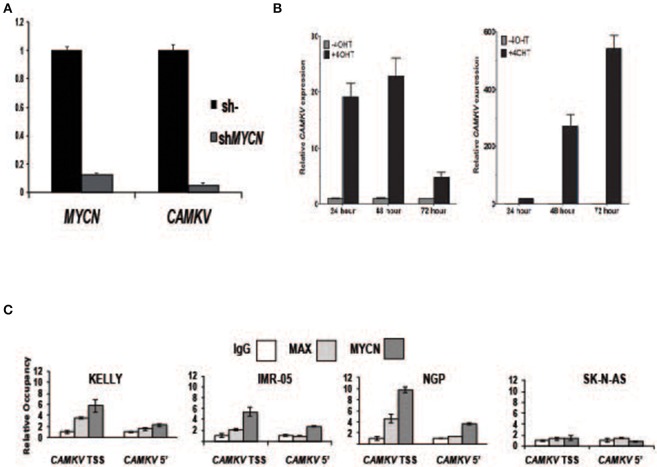
MYCN protein directly controls *CAMKV* transcription. **(A)** MYCN depletion by shRNA in the *MYCN* amplified IMR-05 cell line causes a reduction in *CAMKV* mRNA. Error bars represent standard deviation of technical triplicates, *p* = < 0.005 for both *MYCN* and *CAMKV* transcript depletions. **(B)** MYCN-inducible SK-N-AS (left) and SHEP (right) were treated with tamoxifen and expression of *CAMKV* was analyzed following 24, 48, and 72 h of treatment. Error bars represent standard deviation of technical triplicates, *p* < 0.01 for all changes in *CAMKV* expression. **(C)** Chromatin immunoprecipitation followed by qPCR was used to confirm the presence of MYCN and MAX proteins at the *CAMKV* TSS and a downstream (5') site in the *CAMKV* locus in three *MYCN* amplified cell lines (Kelly, IMR-05, and NGP) and the non-amplified cell line SK-N-AS. Error bars represent standard deviation of technical triplicates.

To investigate whether MYCN protein regulates *CAMKV* expression, we transiently depleted MYCN from *MYCN* amplified IMR-05 cells using shRNA and saw a significant reduction in *CAMKV* mRNA levels (Figure 4A, *p* < 0.005), suggesting that MYCN is required for *CAMKV* transcription in *MYCN* amplified neuroblastoma cells. Next, to determine if MYCN protein could drive *CAMKV* transcription, we used SHEP and SK-N-AS cells with a tamoxifen-inducible construct to translocate MYCN to the nucleus in cells that do not normally express MYCN. Upon MYCN translocation, we observed a significant increase in *CAMKV* mRNA expression in these cells, indicating that MYCN is also sufficient to drive *CAMKV* transcription (Figure 4B, *p* < 0.01). The MYCN ChIP-seq data revealed that the *CAMKV* TSS has a MYCN-occupied E-box motif. To confirm this occupancy, we designed primers to amplify the locus containing the E-box motif and a 5′ locus that did not have an E-box motif or an occupancy peak in the ChIP-seq data sets. We performed ChIP-qPCR in three *MYCN* amplified cell lines (KELLY, NGP, and IMR-05) and the non-amplified cell line, SK-N-AS. These data confirmed that the MYCN and MAX proteins occupy the E-box motif at the TSS in *MYCN* amplified cell lines and not in SK-N-AS cells and do not have significant binding to the 5′ locus (Figure 4C).

### CAMKV Is a Plasma Membrane-Bound Protein in Neuroblastoma Cells

**Figure 5:**
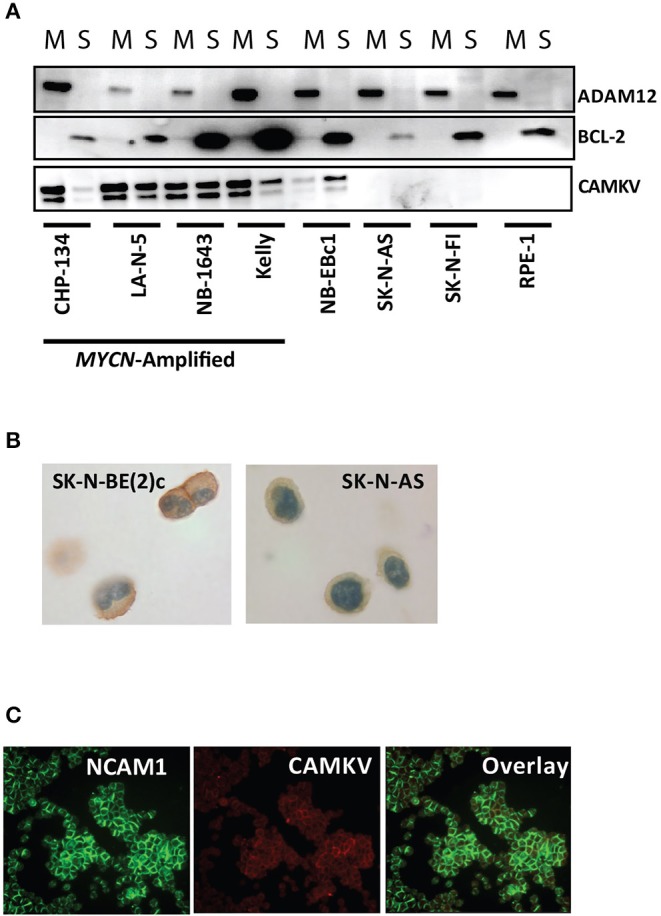
CAMKV protein is expressed on the plasma membrane. **(A)** Membrane fractionation of the *MYCN* amplified neuroblastoma cell lines CHP-134, LA-N-5, NB-1643, and KELLY, the non-amplified cell lines NB-Ebc1, SK-N-AS, and SK-N-FI, and immortalized retinal pigment epithelial cells (RPE-1). ADAM12 is a marker for the membrane fraction (M) and BCL-2 is a marker for the soluble fraction (S). **(B)** CAMKV protein expression detected by immunohistochemistry on cell plugs from the *MYCN* amplified SK-N-BE (2)c and non-amplified SK-N-AS neuroblastoma cell lines. **(C)** CAMKV protein expression detected by immunofluorescence on the *MYCN* amplified NGP neuroblastoma cell line. NCAM1 is used as a marker for the plasma membrane.

CAMKV is known to localize to neurite outgrowths in the brain and thus is predicted to be a membrane-bound protein. Furthermore, CAMKV is predicted to have a transmembrane domain (43). To confirm transmembrane localization in neuroblastoma, we first performed a membrane extraction in four *MYCN* amplified neuroblastoma cell lines [KELLY, CHP-134, LA-N-5, and SK-N-BE (2)c], three non-amplified neuroblastoma cell lines (NB-Ebc1, SK-N-AS, SK-N-FI) and RPE-1 cells. We used ADAM12 as a marker for the membrane fraction (44) and BCL-2 as a marker for the soluble fraction of cells (45). CAMKV protein was present in the membrane fraction of the four *MYCN* amplified neuroblastoma cell lines and NB-EBc1 cells [which have known low basal MYCN protein expression without locus amplification (18)] (Figure 5A). Next, we created cell plugs from both *MYCN* amplified and non-amplified neuroblastoma cell lines and performed immunocytochemistry for CAMKV. This revealed CAMKV protein to be expressed and membrane-bound in *MYCN* amplified SK-N-BE (2)c cells but not expressed in non-amplified SK-N-AS cells (Figure 5B). Finally, the membrane localization of CAMKV was confirmed in *MYCN* amplified NGP neuroblastoma cells by immunofluorescence (Figure 5C). We used NCAM1 (CD56), an established cell surface molecule expressed in neuroblastoma as a marker for the plasma membrane (32, 46) and found NCAM1 and CAMKV proteins co-localize on the plasma membrane of NGP cells. Together, these data show that CAMKV is a membrane-bound protein in *MYCN* amplified neuroblastoma with the potential to be targeted using a CAMKV-specific biologic.

## Discussion

Here we present a computational pipeline to discover cell-surface immunotherapeutic targets in *MYCN* amplified neuroblastoma. *MYCN* amplification status is routinely tested for in all newly diagnosed neuroblastoma patients (1), and we sought to discover therapeutic targets that could utilize *MYCN* amplification as a biomarker for efficacy. The intersection of the most differentially overexpressed genes in tumors with the highest *MYCN* expression and genes predicted to be located in the plasma membrane resulted in 14 potential cell-surface targets in *MYCN* amplified neuroblastoma tumors, 11 of which have TSS-proximal E-boxes. Chromatin immunoprecipitation with sequencing (ChIP-seq) in *MYCN* amplified cell lines revealed that 4 of these TSS-proximal E-boxes were bound by MYCN protein. Two of these genes had low or absent expression in normal tissues outside of the brain, and *CAMKV* had the highest differential expression in *MYCN* amplified tumors and correlated with *MYCN* expression across all tumors. Perhaps not surprisingly due to the neural lineage specificity of *MYCN* expression, all candidate immunotherapeutic targets identified here showed expression in several brain tissues, obviating cellular immunotherapeutic or small molecule targeting approaches.

*CAMKV* encodes a pseudokinase that has sequence similarity to the calmodulin kinase family of proteins but lacks the essential residues required for kinase activity. *CAMKV* is present post-natally in the mammalian forebrain and is associated with vesicles in axons and dendrites. Recombinant *CAMKV* was found to bind calmodulin in the presence of calcium but lacks kinase activity with a sample substrate (47). CAMKV appears to be a substrate of CDK5 in the developing brain and functions in regulating dendritic spine maintenance (48). Remarkably, *CAMKV* is one of a very limited number of MYCN target genes whose protein product is localized in the plasma membrane and shows significant differential expression compared to normal non-central nervous system tissues. We previously reported that MYCN mediates *GPC2* expression in neuroblastoma and that *GPC2* was overexpressed in neuroblastomas that have 7q gains, where the *GPC2* locus is. Due to high *GPC2* expression in neuroblastomas with 7q gain and diploid *MYCN, GPC2* was not one of the genes to be differentially overexpressed when comparing *MYCN* amplified to non-amplified tumors. Here, we nominate CAMKV as an immunotherapeutic target for *MYCN* amplified neuroblastoma, and suggest pursuing the identification of specific human scFv binders to this protein for the creation of antibody-drug conjugate therapeutics that should not cross the blood-brain barrier.

## Data Availability Statement

The datasets generated for this study can be found in the NCBI Gene Expression Omnibus (GEO) database (GSE138295 and GSE94782).

## Author Contributions

RS, PR, and JM: conceptualization. RS, JR, PR, and JM: methodology. JR, PR, and KR: formal analysis. RS, KH, JR, PR, KB, ML, and LH: investigation. DM, TB, and BP: pathology. RS, JR, and JM: funding acquisition and writing. RS and JM: supervision.

### Conflict of Interest

The authors declare that the research was conducted in the absence of any commercial or financial relationships that could be construed as a potential conflict of interest.

## Supplementary Material

Figure S1 Additional MYCN-target genes identified in the validation Chromatin immunoprecipitation experiments. ChIP-Seq tracks from 5 genes bound by MYCN at transcription start site-proximal E-boxes in the *MYCN* amplified COG-N-415, LA-N-5 and NB-1643 cell lines.

Figure S2 *CAMKV* expression in cancers and normal tissues. **(A)** RNA sequencing data from 150 neuroblastoma tumors and normal tissues in the GTEx project were queried for *CAMKV* **(B)** RNA sequencing data obtained from normal tissues in the GTEx project, and tumors from the TARGET program (including 150 neuroblastomas), PNOC, CBTTC, TCGA and the GEO database (GSE60052, small cell lung cancer) were queried for *CAMKV* RNA expression. For both plots, RNA expression values were FPKM-normalized and log2-transformed. Data are represented by boxplots of the median plus the first and third quartiles. Dots represent outliers.

Table S1 Gene lists from microarray or RNAseq analysis for MYCN high tumors and transmembrane domain-containing gene products.
