# Supplementary figures and images for: CAMKV Is a Candidate Immunotherapeutic Target in MYCN Amplified Neuroblastoma
Source: Front Oncol. 2020 Mar 6;10:302. doi: 10.3389/fonc.2020.00302 (PMC7069022; doi:10.3389/fonc.2020.00302)

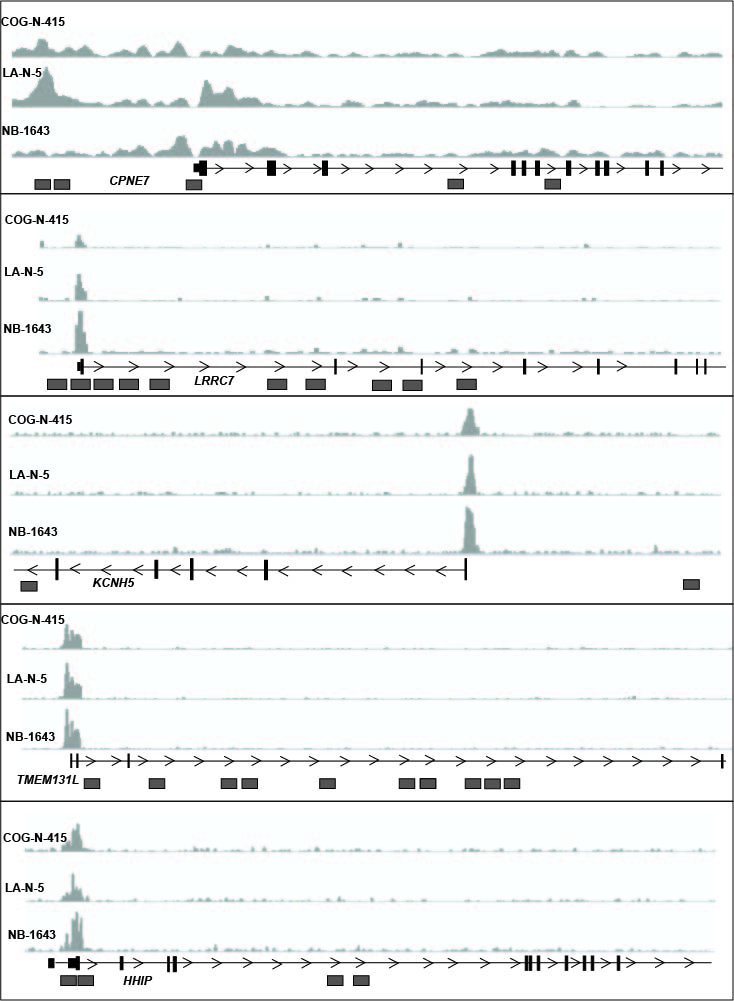

Supplement: Figure S1 — Additional MYCN-target genes identified in the validation Chromatin immunoprecipitation experiments. ChIP-Seq tracks from 5 genes bound by MYCN at transcription start site-proximal E-boxes in the MYCN amplified COG-N-415, LA-N-5 and NB-1643 cell lines. [file Image_1.jpg]

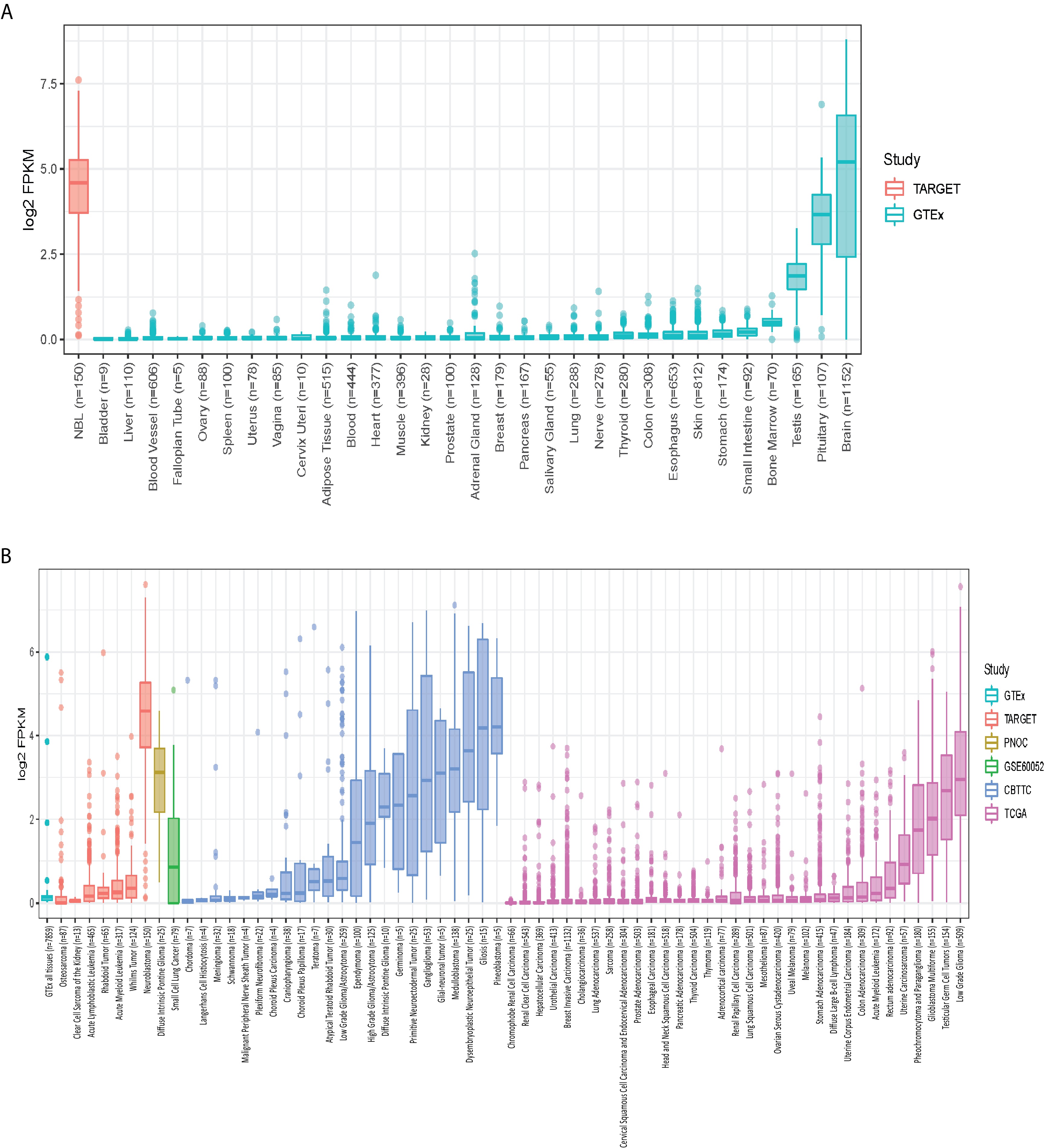

Supplement: Figure S2 — CAMKV expression in cancers and normal tissues. (A) RNA sequencing data from 150 neuroblastoma tumors and normal tissues in the GTEx project were queried for CAMKV (B) RNA sequencing data obtained from normal tissues in the GTEx project, and tumors from the TARGET program (including 150 neuroblastomas), PNOC, CBTTC, TCGA and the GEO database (GSE60052, small cell lung cancer) were queried for CAMKV RNA expression. For both plots, RNA expression values were FPKM-normalized and log2-transformed. Data are represented by boxplots of the median plus the first and third quartiles. Dots represent outliers. [file Image_2.JPEG]
